# Data for oropharyngeal leak pressure, peak inspiratory pressure, and gastric tube insertion success rate of supraglottic airway devices in laparoscopic surgeries (A network meta-analysis dataset)

**DOI:** 10.1016/j.dib.2019.104852

**Published:** 2019-11-21

**Authors:** Sang Won Yoon, Hyun Kang, Geun Joo Choi, Choongun Ryu, Yong Hee Park, Chong Wha Baek, Yong Hun Jung, Young Cheol Woo

**Affiliations:** Chung-Ang University College of Medicine, South Korea

**Keywords:** Laparoscopy, Laryngeal masks, Network meta-analysis, Respiratory mechanics

## Abstract

This article presents dataset of network meta-analysis (NMA) and systemic review, entitled, *Comparison of supraglottic airway devices in laparoscopic surgeries: A network meta-analysis* Yoon SW et al., 2019. The data tables demonstrate numeric values for endpoints: oropharyngeal leak pressure (OLP) before and after pneumoperitoneum, peak inspiratory pressure (PIP) before and after pneumoperitoneum, and gastric tube insertion success rate for each supraglottic airway device (SAD). All relevant randomized controlled trials published up to 31 March 2018 were collected from MEDLINE, EMBASE, Web of Science, Cochrane Central Register of Controlled Trials (CENTRAL), and Google Scholar databases. 26 studies with a total of 2142 patients that included eight different SADs were included. The data described in this article are available as a supplementary file.

**Table:** Specifications Table

Subject	Anesthesiology and Pain Medicine
Specific subject area	Supraglottic airway devices, oropharyngeal leak pressure
Type of data	Table, excel file
How data were acquired	All relevant randomized controlled trials published up to 31 March 2018 were collected from MEDLINE, EMBASE, Web of Science, Cochrane Central Register of Controlled Trials (CENTRAL), and Google Scholar databases.
Data format	Raw
Parameters for data collection	All relevant randomized controlled trials published up to 31 March 2018.
Description of data collection	Data were collected from MEDLINE, EMBASE, Web of Science, Cochrane Central Register of Controlled Trials (CENTRAL), and Google Scholar databases.
Data source location	City/Town/Region: Seoul Country: Republic of Korea
Data accessibility	Dataset available as an Excel file accompanying this article as a supplementary file
Related research article	Author's name: Yoon SW, Kang H, Choi GJ, Ryu C, Park YH, Baek CW, Jung YH, Woo YC Title: Comparison of supraglottic airway devices in laparoscopic surgeries: A network meta-analysis. Journal DOI: 10.1016/j.jclinane.2018.12.044.

**Table:** 

**Value of the Data** • This dataset provides a comprehensive assessment of supraglottic airway devices used in laparoscopy. • Oropharyngeal leak pressure, peak inspiratory pressure, and gastric tube insertion success rate were compared among supraglottic airway devices before and after pneumoperitoneum. • This data can be helpful for physicians choosing different supraglottic airway devices in various medical settings. • The obtained data can be used in further studies to compare supraglottic airway devices not included in this network meta-analysis.

## 1 Data

110 studies from MEDLINE, EMBASE, Web of Science, CENTRAL, Google Scholar database search and manual search were evaluated and after omitting duplicates, 103 studies remained. Of these, full texts of the 26 were evaluated in detail and remaining studies were discharged because they were out of our interest. Therefore, this NMA includes 26 studies with 2142 patients that assessed eight different SADs. The characteristics of the 26 studies are summarized in Table 1 of the primary research article: *Comparison of supraglottic airway devices in laparoscopic surgeries: A network meta-analysis Yoon SW* et al.*, 2019.*

In laparoscopic surgeries where airway pressure rises, high oropharyngeal leak pressure (OLP) is crucial in maintaining tidal volume without leakage. Also, low peak inspiratory pressure (PIP) protects patients' lungs from barotrauma and therefore ensures safe ventilation. Therefore, we collected these data from each RCTs to compare different SADs and rank which SAD is the most effective in laparoscopy. The numeric value of OLP before and after pneumoperitoneum, PIP before and after pneumoperitoneum, and gastric tube insertion success rate for each supraglottic airway device (SAD) are presented in Tables 1–5 (available in accompanying supplementary Excel Spreadsheet file).

## 2 Experimental design, materials, and methods

A. Inclusion and Exclusion Criteria

Data of randomized controlled trials (RCTs) comparing two or more SADs for laparoscopic surgery were included in this dataset. Patients were restricted to adults that received laparoscopic surgery under general anesthesia. 8 different SAD were as follows:1) laryngeal mask airway Classic (LMA; LMA-C), 2) LMA ProSeal (LMA-P), 3) LMA Supreme (LMA-S), 4) i-gel, 5) Cobra Perilaryngeal Airway (CobraPLA), 6) Streamlined Liner of the Pharynx Airway (SLIPA), 7) laryngeal tube suction (LTS), and 8) Ambu AuraGain. These SADs were compared with other SADs and outcomes were OLP, PIP before and after pneumoperitoneum and success rate of gastric tube insertion. Two studies [1,2] did not specify the type of SADs that were evaluated; we classified them as LMA-C.

Data that did not report the outcomes of interest and those that were not RCTs were excluded in this data. There were neither language limitations nor date restrictions in our data.

B. Data Extraction

Two authors independently extracted and entered relevant data from included and they were cross-checked. Attempts were made to contact the study authors to obtain the relevant information in cases of missing or incomplete data. Data that were presented as figures and graphs [3,4] were extracted as numbers using open source software Plot Digitizer (version 2.6.8; http://plotdigitizer. sourceforge.net). The extracted data from figures are highlighted in blue in Tables 1–4 of supplementary Excel Spreadsheet file.

C. Risk of Bias Assessment

The quality of data obtained from included RCTs was assessed using the tool of ‘risk of bias’ according to Review Manager (version 5.3, The Cochrane Collaboration, Oxford, UK). The following potential sources of bias were evaluated: sequence generation, allocation concealment, blinding of participants or outcome assessor, incomplete data, and selective reporting. Table 2 of the primary research article: *Comparison of supraglottic airway devices in laparoscopic surgeries: A network meta-analysis Yoon SW* et al.*, 2019* describes risk of bias assessment.
